# TIM-3 Does Not Act as a Receptor for Galectin-9

**DOI:** 10.1371/journal.ppat.1003253

**Published:** 2013-03-21

**Authors:** Judith Leitner, Armin Rieger, Winfried F. Pickl, Gerhard Zlabinger, Katharina Grabmeier-Pfistershammer, Peter Steinberger

**Affiliations:** 1 Division of Immune Receptors and T Cell Activation, Institute of Immunology, Center for Pathophysiology, Infectiology and Immunology, Medical University of Vienna, Vienna, Austria; 2 Division of Immunology, Allergy and Infectious Diseases, Department of Dermatology, Medical University of Vienna, Vienna, Austria; 3 Institute of Immunology, Center for Pathophysiology, Infectiology and Immunology, Medical University of Vienna, Vienna, Austria; University of Pennsylvania, United States of America

## Abstract

T cell immunoglobulin and mucin protein 3 (TIM-3) is a type I cell surface protein that was originally identified as a marker for murine T helper type 1 cells. TIM-3 was found to negatively regulate murine T cell responses and galectin-9 was described as a binding partner that mediates T cell inhibitory effects of TIM-3. Moreover, it was reported that like PD-1 the classical exhaustion marker, TIM-3 is up-regulated in exhausted murine and human T cells and TIM-3 blockade was described to restore the function of these T cells. Here we show that the activation of human T cells is not affected by the presence of galectin-9 or antibodies to TIM-3. Furthermore, extensive studies on the interaction of galectin-9 with human and murine TIM-3 did not yield evidence for specific binding between these molecules. Moreover, profound differences were observed when analysing the expression of TIM-3 and PD-1 on T cells of HIV-1-infected individuals: TIM-3 was expressed on fewer cells and also at much lower levels. Furthermore, whereas PD-1 was preferentially expressed on CD45RA^−^CD8 T cells, the majority of TIM-3-expressing CD8 T cells were CD45RA^+^. Importantly, we found that TIM-3 antibodies were ineffective in increasing anti-HIV-1 T cell responses *in vitro*, whereas PD-L antibodies potently reverted the dysfunctional state of exhausted CD8 T cells. Taken together, our results are not in support of an interaction between TIM-3 and galectin-9 and yield no evidence for a functional role of TIM-3 in human T cell activation. Moreover, our data indicate that PD-1, but not TIM-3, is a promising target to ameliorate T cell exhaustion.

## Author Summary

Inhibitory costimulatory receptors are a hallmark of exhausted T cells, which accumulate during chronic infection with viruses like HIV-1. Recently, TIM-3 was described as functional receptor on exhausted human T cells. Galectin-9 was reported as an inhibitory ligand for TIM-3 on murine T cells, but it was not known whether galectin-9 has a role in human T cell activation processes. We have found that the activation of human T cells is not affected by the presence of galectin-9 or antibodies to TIM-3. Furthermore, we demonstrate that galectin-9 does not serve as a ligand of human or murine TIM-3. Analysis of T cells of HIV-infected individuals regarding the expression of TIM-3 and PD-1 demonstrates that TIM-3 is expressed on fewer cells and also at much lower levels. In fact, TIM-3 expression characterizes a T cell population that is distinct from the PD-1 expressing exhausted T cells. Our results indicate that PD-1, but not TIM-3, is a promising target to ameliorate T cell exhaustion.

## Introduction

Inhibitory costimulatory signals play a decisive role in the outcome of T cell responses and there is an ever-growing number of pathways that have been implicated in such processes. The successful clinical use of CTLA-4 antibodies to enhance anti-melanoma T cell responses underlines the therapeutic potential of antibodies targeting negative costimulatory T cell pathways [1].

During the last years, it has been acknowledged that inhibitory pathways also significantly contribute to the exhausted state of T cells, which results from persistent antigen stimulation in chronic virus infections or cancer. The inhibitory PD-1 was identified as a marker for such dysfunctional T cells and blockade of PD-1 signals - in most cases realized with antibodies to PD-L1 - was shown to revert the dysfunctional state of exhausted T cells [2]–[4]. Peretz et al. have found that co-expression of PD-1 and CD160, another inhibitory receptor, defined a subset with advanced dysfunction [5]. We have recently shown that PD-1 is up-regulated in CD4 and CD8 T cells from HIV-infected individuals, who have impaired immune reconstitution despite successful antiretroviral therapy, indicating that PD-1 blockade might be beneficial also in these patients [6]. PD-1 is also a promising target to improve T cell immunity in cancer patients and clinical trials to assess the safety and efficacy of blocking PD-1 and PD-L1 antibodies have been initiated [7], [8]. Additional inhibitory receptors have been demonstrated to be associated with a dysfunctional phenotype and Blackburn et al. have shown that exhausted T cells express up to seven different inhibitory receptor molecules [9]. Several studies indicate that among these molecules, the T cell immunoglobulin and mucin domain 3 (TIM-3) has an important role in maintaining the dysfunctional state of exhausted T cells, as TIM-3 blockade restored proliferation and cytokine production upon antigenic challenge in these cells [10]–[13]. Consequently, it has been suggested that blocking TIM-3/TIM-3 ligand interactions might be of clinical utility by restoring the function of virus or tumour-specific T cells.

However, TIM-3 appears to be a pleiotropic immune receptor and many aspects of human TIM-3 functions have not been clarified to date. Murine TIM-3 was reported to negatively regulate T cell responses via interacting with galectin-9 [14] but currently it is not known whether human TIM-3 acts as galectin-9 receptor and the functional role of galectin-9 during the activation of human T cells has not been investigated. Furthermore, apart from a study on NY-ESO-1 specific CD8^+^ T cells derived from melanoma patients, the effects of TIM-3 and PD-L1 blockade have not been compared [12].

In this study, we have addressed the functional role of galectin-9 during the activation of human T cells. In addition, we have performed extensive experiments to study the interaction between TIM-3 and galectin-9 molecules of human and mouse origin. Finally, we used Gag/Nef - specific T cells derived from HIV-1-infected individuals as model system to compare the ability of antibodies to TIM-3 and PD-ligands to counter-act T cell dysfunction and exhaustion.

## Results

### Presence of galectin-9 does not inhibit activation or induce cell death in human T cells

**Figure 1:**
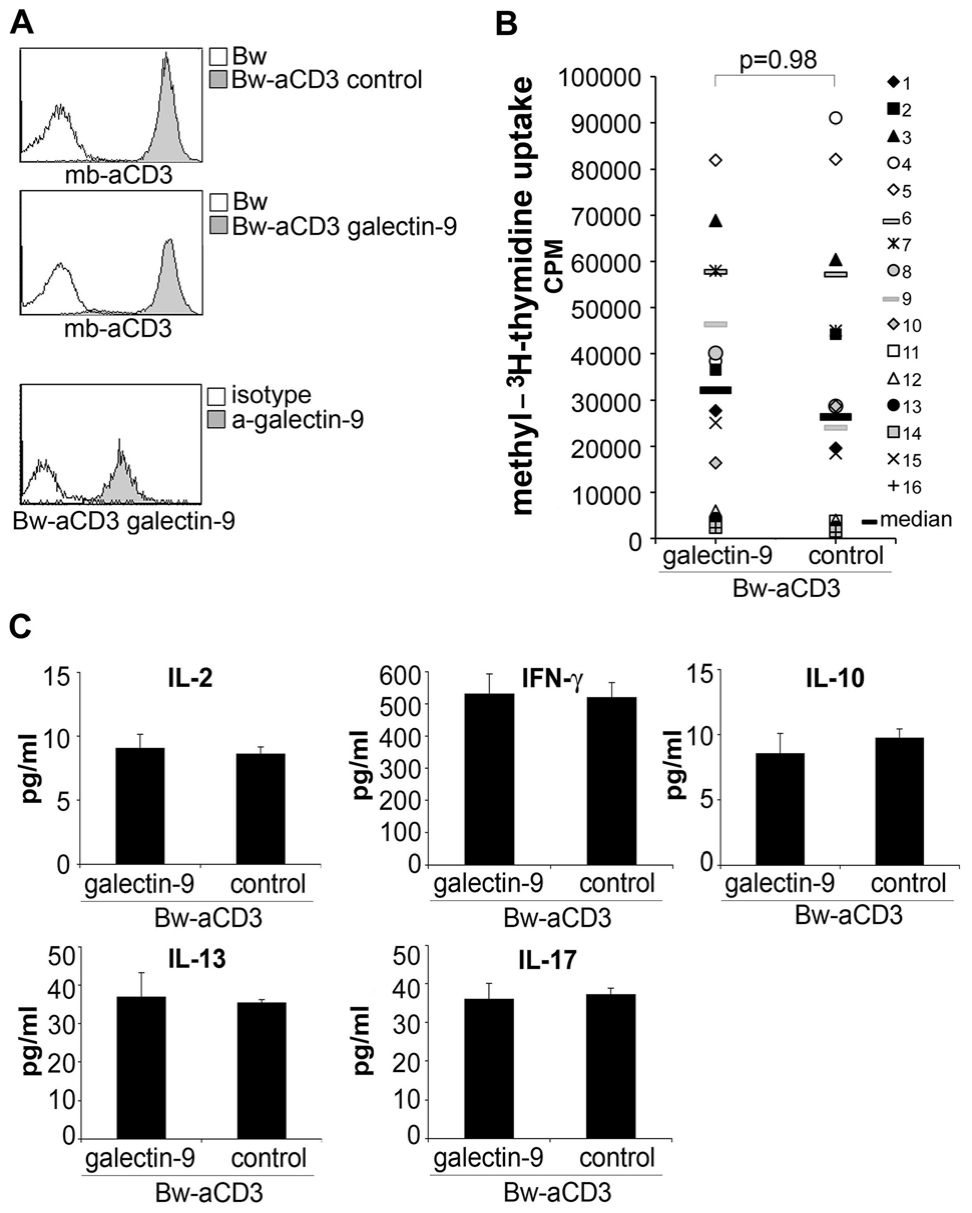
Galectin-9 does not costimulate human T cell proliferation. A) Characterization of T cell stimulator cells. Upper and middle panel: Expression of membrane-bound anti-CD3 antibody fragments (mb-aCD3) on T cell stimulator cells was detected with Dylight-649-labelled goat-anti-mouse IgG (H+L-chain specific) antibodies (grey histograms). Open histogram: reactivity with control Bw cells. Lower panel: T cell stimulator cells engineered to express human galectin-9 were probed with galectin 9 antibodies (grey histogram) or isotype control antibodies (open histogram). B) Human T cells derived from healthy donors were co-cultured with T cell stimulator cells expressing human galectin-9 or no human costimulatory molecule (control). ^3^[H]-methyl-thymidine uptake was assessed following 3 days of co-culture (cpm, counts per minute). The results summarize 16 experiments with different donors. C) Culture supernatant derived from T cells activated with galectin-9 expressing stimulator cells or control stimulator cells was harvested after 48 h and subjected to multiplex cytokine measurement. Data show +/− SD of triplicates from one experiment representative for 16 independently performed.

Previously, it was reported that galectin-9 binds murine TIM-3, thereby negatively regulating murine T cells [14]. Since little is known regarding the functional role of galectin-9 on human T cells, we performed experiments in which T cells were activated in the presence of human galectin-9. In these experiments, we used our previously described system of T cell stimulator cells that can stimulate human T cells via membrane-bound anti-CD3 antibody fragments [15]. Control stimulator cells and stimulator cells expressing high levels of human galectin-9 were established and analyzed for expression of anti-CD3 antibodies and galectin-9 (Figure 1A). Human T cells were co-cultured with these stimulator cells and T cell proliferation and cytokine production were assessed. As shown in Figure 1B, the presence of galectin-9 during stimulation did not result in a reduced proliferative response in human T cells. By contrast, stimulator cells expressing the well-established inhibitory ligand PD-L1, used under the same conditions, significantly reduced human T cell proliferation, whereas activating costimulatory ligands (CD80; CD86) strongly enhanced T cell proliferation when expressed on the stimulator cells (Figure S1A). Furthermore, we found that the production of cytokines was also not affected by human galectin-9 (Figure 1C). Whereas galectin-9 was reported to induce cell death in murine T cells [14], we could not observe such effects in human T cells: In T cells activated with T cell stimulator cells expressing galectin-9 there was no increase in apoptotic T cells. By contrast, the number of T cells undergoing apoptosis was higher when T cell stimulator cells expressing human FasL were used to activate T cells (Figure S1B).

### TIM-3 does not interact with galectin-9

**Figure 2:**
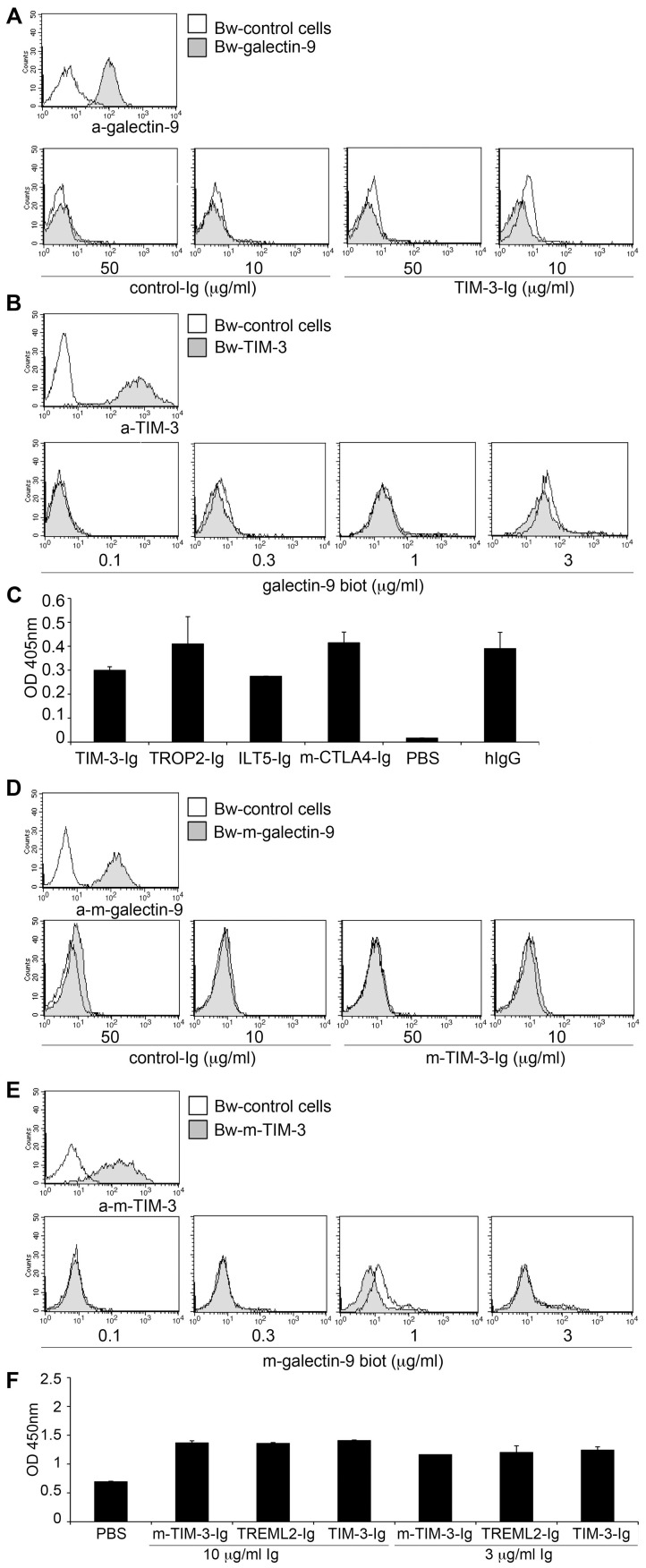
TIM-3 does not interact with galectin-9. A, D) Bw control cells (open histograms) or Bw cells transduced to express human (A) or murine (D) galectin-9 (grey histograms) were probed with galectin-9 antibodies or with immunoglobulin fusion proteins representing human TIM-3 (TIM3-Ig; A) or murine TIM-3 (m-TIM-3-Ig; D) or a control fusion protein (B7-H3-Ig). Bound antibodies were detected with PE-labelled donkey-anti-goat antibodies. Immunoglobulin fusion proteins were detected with PE-labelled goat antibodies to the Fc-part of human IgG. B, E) Bw control cells (open histogram) or Bw cells transduced to express human (B) or murine (E) TIM-3 (grey histograms) were probed with appropriate anti-TIM-3 mAbs or with biotin-labelled recombinant human (B) or murine (E) galectin-9. Bound anti-human TIM-3 mAb and anti-murine TIM-3 mAb were detected with PE-labelled goat-anti-mouse IgG and APC-labelled goat-anti-rat IgG, respectively. SA-PE was used as secondary reagent for biotin-labelled recombinant human and murine galectin-9. C, F) Recombinant human (C) or murine (F) galectin-9 was immobilized on ELISA plates and probed with immunoglobulin fusion proteins (Ig) representing the extra-cellular domain of human TIM-3 (TIM-3-Ig; C) or murine TIM-3 (m-TIM-3-Ig; F) at the indicated concentrations. HRP-conjugated goat-anti-human IgG-Fc-specific antibodies were used for detection. Binding buffer only (PBS) or the indicated immunoglobulin fusion proteins were used as controls. All binding experiments were repeated three times with similar outcome.

Previous studies reported that the inhibitory effect of galectin-9 on murine T cells is mediated at least in part by TIM-3 [14], [16]–[18]. Although galectin-9/TIM-3 interaction was reported to protect human CD4 T cells against HIV-1 infection [19], to date there are no reports demonstrating a specific binding of human galectin-9 to human TIM-3. Therefore, we performed a series of experiments to study a potential interaction between these two molecules. In the first set of experiments, we probed Bw cells expressing human galectin-9 on their surface with an immunoglobulin fusion protein representing the extra-cellular domain of TIM-3 fused to the Fc-part of human IgG_1_ (TIM-3-Ig) or with B7-H3-Ig used as control. In these experiments, a specific interaction between cell-expressed human galectin-9 and TIM-3-Ig was not observed (Figure 2A). Binding of CD80-Ig to CD28 expressing Bw cells was readily detected under the same experimental conditions even to cells that expressed low levels of CD28 (Figure S2A). In addition, we probed Bw cells expressing human TIM-3 or Bw-control cells with biotin-labelled recombinant human galectin-9 at different concentrations. When used at higher concentrations galectin-9 was found to interact with parental Bw cells. Galectins are known to bind to β-galactose containing glyco-conjugates and thus the interaction with β-galactose containing glycoproteins expressed on the surface of Bw cells is likely to account for this binding. Importantly, this interaction was independent of TIM-3, since the binding signals were not enhanced on cells expressing high levels of human TIM-3 on their surface (Figure 2B). In addition, we performed binding studies where galectin-9 antibodies were used for detection. Also in these experiments we did not observe increased binding to the TIM-3 expressing cells (Figure S2B). Finally, we performed an ELISA, where immobilized recombinant human galectin-9 was probed with an immunoglobulin fusion protein representing the extra cellular domain of human TIM-3 (TIM-3-Ig). For control purposes, the binding of ILT5-Ig, mouse-CTLA4-Ig, TROP2-Ig, TREML2-Ig from the same manufacturer and human IgG was analyzed in parallel. Also in these experiments we did not observe a specific interaction between human TIM-3 and galectin-9, since compared to the control fusion proteins, the binding signals observed with TIM-3-Ig were not higher (Figure 2C). Furthermore, when we performed ELISA binding experiments that cover a broad concentration range (0.3–9 µg/ml) we did not obtain higher binding signals with TIM-3-Ig compared to murine CTLA4-Ig that was used as a control (Figure S2C). In these experiments, we also included TIM-3-Ig and a control fusion protein (EpCam-Ig) produced in our laboratory. Compared to the commercial fusion proteins (all expressed in NSO-cells), the ELISA signal obtained with our fusion proteins was generally much weaker, which could indicate that the NSO-expressed fusion proteins harbour modifications that account for the galectin-9 binding that was observed with all commercial preparations tested. Importantly, with both types of TIM-3-Ig preparations we did not observe higher binding signals compared to the respective control proteins (Figure S2C). Since receptor-ligand interactions are usually conserved between mice and men, we also performed a series of experiments to analyze the interaction between murine TIM-3 and galectin-9. However, as expected from the experiments with their human orthologues and in contrast to a previous report [14], a specific interaction between cell expressed murine galectin-9 and murine TIM-3-Ig fusion proteins was not detected (Figure 2D). Likewise, membrane-resident murine TIM-3 did not bind soluble recombinant murine galectin-9, and ELISA experiments did also not yield evidence for a specific interaction between murine TIM-3 and galectin-9 (Figure 2E, F). Binding studies in the presence of calcium ions were also performed with the same outcome (data not shown).

### TIM-3 antibodies do not affect human T cell activation

**Figure 3:**
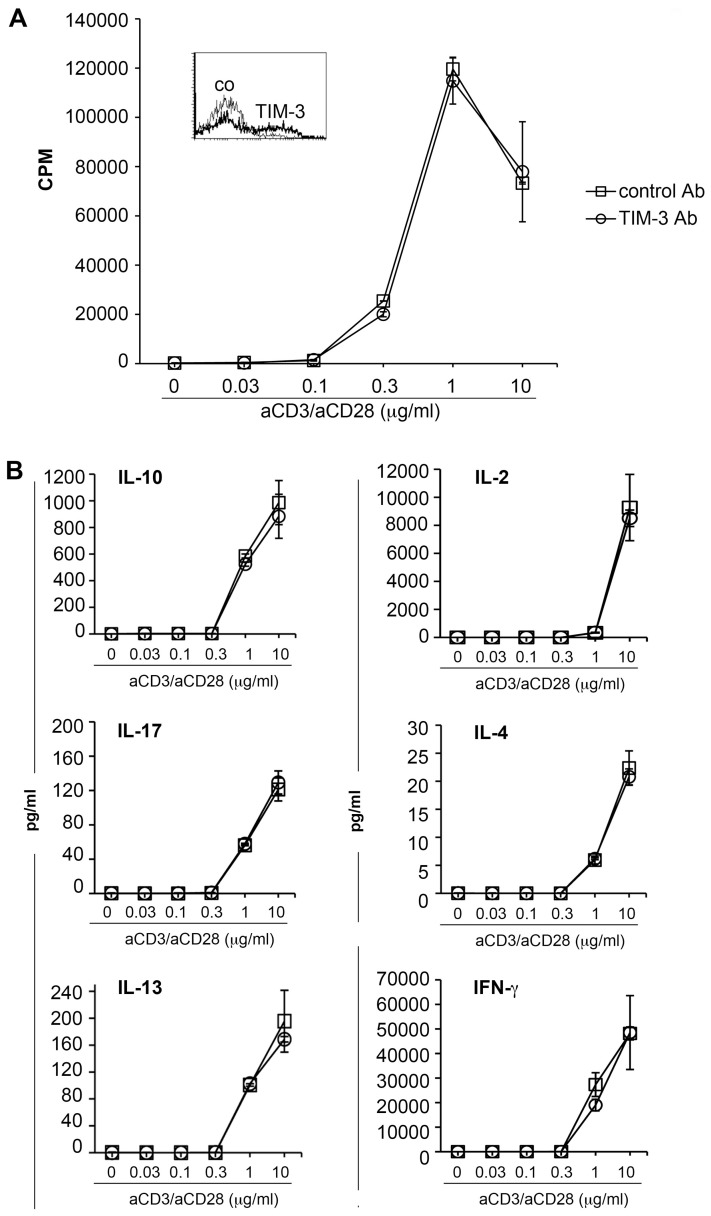
TIM-3 antibodies do not affect human T cell activation. CD4 T cells were stimulated with plate-bound antibodies to CD3 and CD28 immobilized at the indicated concentration. The expression of TIM-3 (bold line; isotype control: thin line) following activation is shown (inset). TIM-3 antibody (clone 2E2) or control antibody (both at a final concentration of 10 µg/ml) were added to the cultures and following 48 h of stimulation, culture supernatants were harvested and methyl-^3^[H]-thymidine was added to the cultures. A) Upon 18 h of additional culturing, cells were harvested and methyl-^3^[H]-thymidine uptake was measured to assess T cell proliferation. B) The cytokine concentration in the supernatants was measured using a Luminex-based multiplex assay. The results of triplicate measurement of one experiment representative for four experiments are shown.

In a next set of experiments, we assessed the function of TIM-3 in human T cell activation using an antibody (clone 2E2) that was previously described and commonly referred to as blocking TIM-3 antibody [10]–[12], [19]–[23]. CD4 T cells were stimulated with plate-bound antibodies to CD3 and CD28, and proliferation and cytokine secretion were measured following 48 hours of stimulation. Whereas Hastings et al. reported that TIM-3 antibody 2E2 enhanced cytokine production, but not T cell proliferation under these experimental conditions [20], we did not observe any functional effects of clone 2E2 as neither proliferation nor cytokine production was affected by the addition of this TIM-3 antibody (Figure 3).

### PD-L blockade but not TIM-3 antibodies increase allogeneic responses of human Th1 cells

**Figure 4:**
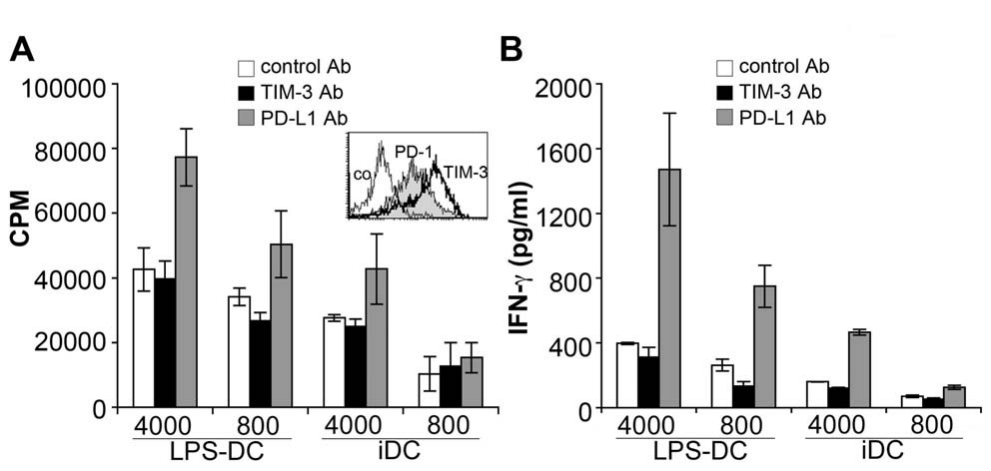
PD-L blockade but not TIM-3 antibodies increase allogeneic responses of human Th1 cells. Human MNC were stimulated under Th1 polarizing conditions. The expression of PD-1 (grey histogram) and TIM-3 (bold line) following Th1 polarization is shown (inset). Subsequently, polarized cells were co-cultured with immature and mature allogeneic DC (iDC and LPS-DC, respectively; numbers of DC/well are indicated) in presence of antibodies to TIM-3 or to PD-L1. Proliferation (A) and IFN-γ production (B) was measured following 6 days of cultures. The results of triplicate measurements of one experiment representative for four experiments are shown.

Since CD4 T cells express little TIM-3 during primary stimulation we also performed experiments where the effects of TIM-3 antibody 2E2 were assessed on T cells harbouring TIM-3 at a high density. PBMC were stimulated with antibodies to CD3 and CD28 under Th1 polarizing conditions. In line with previous reports this treatment resulted in T cells with high surface TIM-3 expression (Figure 4A; inset). However, presence of TIM-3 antibody 2E2 during the stimulation of such T cells with allogeneic DC again did not result in an increase in proliferation or cytokine production. By contrast, we found that a blocking PD-L1 antibody strongly enhanced proliferation and cytokine production in these experiments (Figure 4A, B).

### TIM-3 expression characterizes a terminally differentiated T cell subset in chronically HIV-1-infected patients

**Figure 5:**
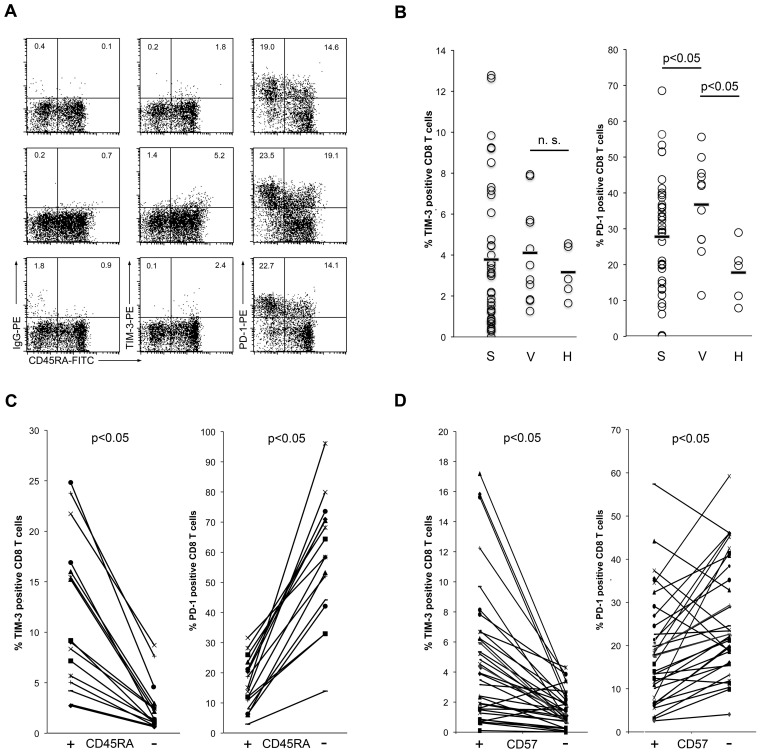
TIM-3 expression on CD8 T cells of HIV-1-infected individuals. A) TIM-3 and PD-1 expression on CD8 T cells from HIV-1-infected individuals. The percentage of positive cells in the CD45RA^+^ and CD45RA^−^ subsets are indicated. Correct assessment of TIM-3 and PD-1 positive cells was ensured by isotype control antibody (IgG-PE) staining for each sample. Representative samples from a suppressed (upper panels) and from viremic patients (middle and lower panels) are shown. B) Percentage of TIM-3^+^ (left graph) and PD-1^+^ (right graph) CD8 T cells from suppressed (S) and viremic (V) patients and from healthy individuals (H) are shown. Bars indicate median percentage. C) Percentage of TIM-3^+^ (left graph) and PD-1^+^ (right graph) CD8 cells in the CD45RA^+^ and CD45RA^−^ subsets. D) Percentage of TIM-3^+^ (left graph) and PD-1^+^ (right graph) CD8 cells in the CD57^+^ and CD57^−^ subset.

TIM-3 has recently been described as a marker of human T cell exhaustion and it was reported to be expressed at high levels on CD8 T cells of HIV-1-infected patients. Moreover, TIM-3 antibodies were reported to revert the dysfunctional state of T cells, which suggests that TIM-3 has a function in the maintenance of T cell exhaustion. We thus assessed TIM-3 expression on CD8 T cells of viremic HIV-1-infected patients and of patients under antiretroviral therapy with no detectable viral load in peripheral blood (virologically suppressed). For comparison, the expression of PD-1, a well-established marker for human T cell exhaustion, was also analyzed. We found TIM-3 to be expressed on only a very small proportion of CD8 T cells in these individuals, whereas PD-1 was strongly up-regulated (Figure 5A). Importantly, the weak staining signal obtained with the TIM-3 antibodies was not due to a weaker reactivity of the antibody used, since TIM-3 and PD-1 antibodies reacted both very strongly with transductants expressing their cognate antigens (Figure S3). Although the percentage of TIM-3 positive CD8 T cells was slightly higher in HIV-1-infected donors, the difference was not statistically significant, whereas PD-1 was significantly increased in HIV infected patients (Figure 5B). TIM-3 expression did not correlate with disease progression parameters (CD4 cell count and viral load; data not shown). Furthermore, we observed that TIM-3 positive CD8 T cells predominantly belong to the CD45RA^+^ T cell subset, which is in contrast to PD-1 expressing T cells that were mainly CD45RA^−^ (CD45RO^+^; Figure 5C). In respect of TIM-3/CD45RA co-expressing T cells, significantly higher numbers could be found in viremic HIV-1-infected patients than in healthy controls, indicating that there is an enrichment of these cells in patients with uncontrolled chronic HIV-1 infection (Figure S4A). When co-staining with TIM-3 and PD-1 antibodies we also observed little co-expression of these molecules on CD8 cells of viremic HIV patients (Figure S4B). CD45RA^+^T cells are found on both ends of the life cycle of T cells and characterize either naïve T cells or terminally differentiated T cells. Since TIM-3 expression has been linked to chronic viral infection and persistent antigen stimulation shifts the T cell repertoire from naïve to terminally differentiated T cells it is likely that TIM-3/CD45RA^+^ T cells belong to the latter. Thus, we analyzed expression of CD57, a marker for senescent T cells, on TIM-3 positive CD8 T cells. We found that the majority of TIM-3 expressing CD8 T cells indeed co-express CD57 and these cells thus belong to late-differentiated T cells. In line with the differences in CD45RA expression, the percentage of PD-1 expressing cells was significantly lower in CD57-expressing CD8 T cells than in CD57-negative CD8 T cells (Figure 5D). Taken together, these data indicate that compared to PD-1 expressing T cells, the number of TIM-3 expressing T cells is much lower in chronically HIV-1-infected patients. Moreover, we show that TIM-3 and PD-1 characterize different CD8 T cells in these individuals.

### TIM-3-antibodies do not reverse T cell exhaustion

**Figure 6:**
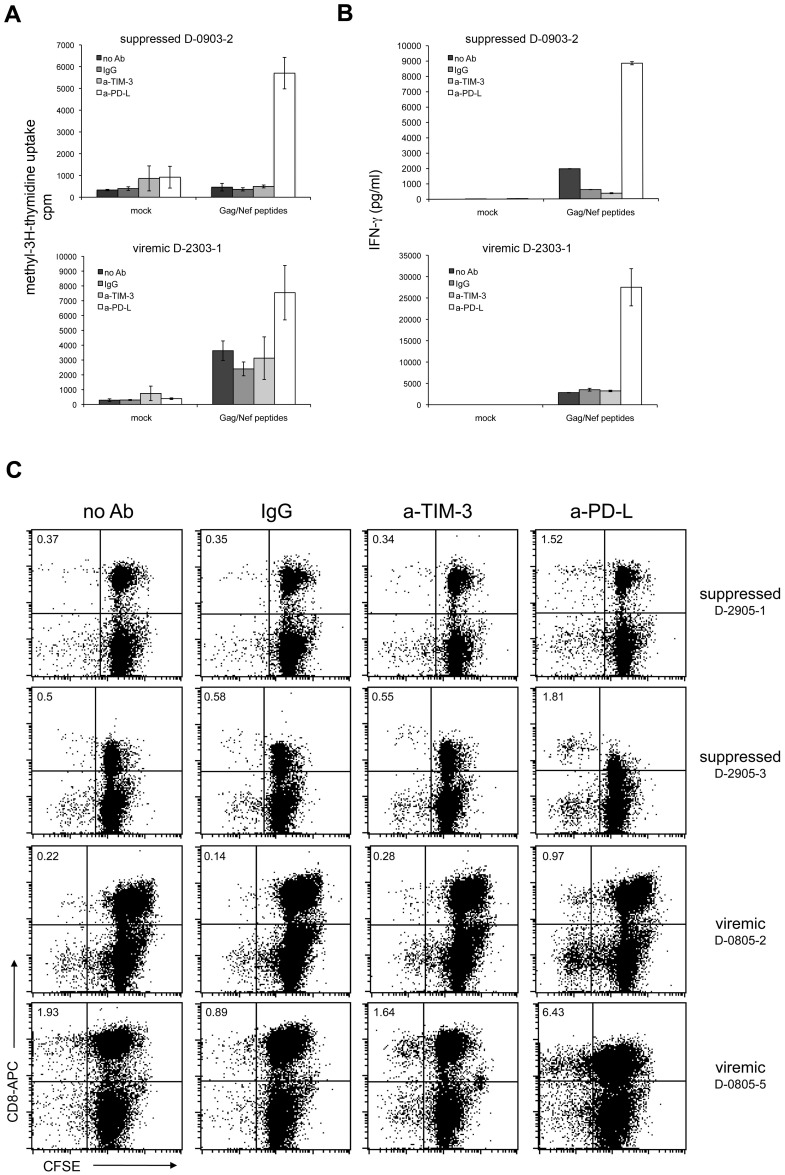
TIM-3 antibodies do not revert T cell exhaustion. PBMC from HIV-1-infected individuals were mock-treated or stimulated with Gag/Nef peptide pools. Control antibodies and antibodies to TIM-3 or PD-L were added as indicated. A) Methyl-^3^[H]-thymidine uptake was measured following seven days of culture. Shown is a representative aviremic and viremic patient. PD-L antibodies (p<0.001; n = 30) but not TIM-3 antibody (p = 0.59; n = 30) significantly enhanced methyl-^3^[H]-thymidine uptake compared to controls. B) INF-γ concentration of day 6 culture supernatants. Shown is a representative aviremic and viremic patient. The INF-γ concentration was significantly higher in stimulation cultures with PD-L antibodies (p<0.05; n = 23) whereas TIM-3 antibody (p = 0.92; n = 23) had no effect. C) CFSE dilution experiments with PBMC from suppressed (upper panels) and viremic patients (lower panels). Cells were analyzed on day 7 of culture and the experiments shown are representative of 22 independently performed (7 samples from viremic and 15 samples from suppressed patients). The number of CFSE^low^ CD8 T cells was significantly higher in stimulation cultures with PD-L antibodies (p<0.05; n = 22), whereas TIM-3 antibodies had no effect (p = 0.99; n = 22). A detailed summary of all experiments is given in table 1.

Several studies have reported that similar to PD-1, TIM-3 also has a functional role in T cell exhaustion associated with HIV-1-infection, but also with other chronic viral infections or cancer [10]–[13], [22]. Most of these studies have used the TIM-3 antibody 2E2 to investigate the functional role of TIM-3 during stimulation of CD8 cells with virus or cancer antigens. We used Gag- and Nef-specific T cells as a model system to compare TIM-3 antibody 2E2 and blocking PD-L antibodies regarding their ability to enhance HIV-1-specific T cell responses *in vitro*. In these experiments we found that PD-L blockade but not TIM-3 antibodies consistently enhanced proliferation measured by methyl-^3^[H]-thymidine uptake as well as IFN-γ production in HIV-1-specific T cells (Figure 6A,B; Table 1). Similar results were obtained in CFSE-dilution experiments: PD-1 blockade strongly increased the numbers of CD8 T cells that had proliferated in response to HIV-1 peptides, whereas TIM-3 antibody 2E2 had no effect (Figure 6C; Table 1).

**Table 1:** Effect of antibodies to TIM-3 and PD-Ligands on T cell responses to HIV-peptides.

methyl-^3^[H]-thymidine uptake		Number of experiments (% of total)	Specific response to HIV-peptides n/(%)	TIM-3 antibody			PD-L antibodies		
				enhanced response	Response versus controls	p-value	enhanced response	Response versus controls	p-value
	total	59	30 (51%)	0 (0%)	1.18	0.60	23 (77%)	3.74	4×10^−8^
	viremic	10 (17%)	4 (40%)	0 (0%)	1.04	n.d.	4 (100%)	5.3	n.d.
	suppressed	49 (83%)	26 (53%)	0 (0%)	1.19	n.d.	19 (73%)	3.5	n.d.
**IFN-γ production**									
	total	38	23 (58%)	2 (9%)*	1.21	0.92	15 (65%)	4.96	0.03
	viremic	12 (32%)	6 (50%)	0 (0%)	0.91	n.d.	3 (50%)	2.29	n.d.
	suppressed	26 (68%)	17 (65%)	2 (11%)	1.29	n.d.	12 (71%)	5.66	n.d.
**CFSE dilution**									
	total	42	22 (52%)	1 (4.5%)**	0.96	0.99	16 (73%)	2.49	0.049
	viremic	19 (45%)	7 (37%)	1 (14%)	0.98	n.d.	7 (100%)	3.03	n.d.
	suppressed	23 (55%)	15 (65%)	0 (0%)	0.95	n.d.	11 (73%)	2.24	n.d.

Specific responses to HIV-peptides: Gag/Nef peptide treated samples that had at least 2-fold higher responses than mock treated samples from the same donor; Enhanced responses: HIV-peptide responses in samples treated with TIM-3/PD-L antibodies were at least 1.5-fold higher than both controls (Gag/Nef peptide samples without antibody or treated with isotype control antibody). Response versus controls: The mean response in presence of TIM-3/PD-L antibodies was divided by the mean response of the controls.

* in two samples the IFN-γ production in presence of TIM-3 antibodies was also more than 1.5-fold lower than in both controls;

** in two samples the number of CFSE^low^ cells in presence of TIM-3 antibodies was also more than 1.5 fold lower than in both controls.

n.d.: not determined.

## Discussion

TIM-3 was identified during an attempt to find new Th1-specific cell surface proteins by screening a large panel of monoclonal antibodies raised against murine Th1 T cell clones and lines. Antibodies that stained Th1 cells, but did not react with Th2 cells were found to interact with TIM-3, which was also highly expressed on cytotoxic CD8 cells [24]. The authors reported that TIM-3 antibodies enhanced experimental autoimmune encephalomyelitis (EAE) and suggested that TIM-3 has a role in autoimmune diseases by regulating macrophage number and activation. However, subsequent studies implicated TIM-3 in tolerance induction by interfering with Th1 proliferation and maintaining Treg function [25], [26]. Interestingly, TIM-3 expression was also reported for innate immune cells, where it was found to promote rather than inhibit immune responses [21]. Taken together, these data attribute a very complex functional role to this molecule, which depending on the site of expression may act to promote or inhibit murine immune responses upon interaction with its ligands.

Zhu et al. described galectin-9 as a binding partner for murine TIM-3 and reported that galectin-9/TIM-3 interaction inhibits Th1-responses in mice [14]. Much less is known regarding the functional role of TIM-3 on human immune cells. Importantly, there are no reports how galectin-9 impacts on the activation of human T cells. Therefore, we have used our T cell stimulators, an experimental system that we have devised for the functional evaluation of human costimulatory or coinhibitory ligands, to address a potential functional role of human galectin-9 on human T cells. We found that presence of galectin-9 during the activation of human T cells does not inhibit T cell responses or induce cell death. Since there are no reports that corroborate a specific interaction for human galectin-9 and human TIM-3, we have performed a series of binding studies to analyze a potential interaction between human galectin-9 and TIM-3. Our data did not yield any evidence for such an interaction. In most cases receptor-ligand interactions are conserved between mice and men, and we thus went on to study a potential interaction of murine TIM-3 with murine galectin-9. Using several different experimental settings, we did not detect a specific interaction between these molecules. This is in apparent contrast to a study by Zhu and coworkers [14]. It should however be stressed that in their study the interaction of surface galectin-9 with TIM-3 has not been analyzed. Rather, TIM-3 fusion proteins were used for binding studies with fixed and permeabilized CHO cells transfected with galectin-9 expression constructs. This unusual type of binding experiments was justified by claiming that according to a previous publication by Rabinovich et al. “transient transfection does not produce expression of galectins on the cell surface” [27]. However, this study focuses on galectin-1 and does not contain any statements or data regarding the cell surface expression of galectins in general. It is evident that a specific interaction of surface or soluble galectin-9 with TIM-3 is a pre-requisite for an involvement of galectin-9 in TIM-3 mediated effects. The results of our study largely rule out such an interaction, suggesting that TIM-3 functions are independent of galectin-9. Su et al. have shown that functional effects of galectin-9 do not differ in wild-type and TIM-3 knockout mice, which would also be consistent with our findings and suggests that galectin-9 functions are independent of TIM-3 [28]. Furthermore although TIM-3 has been implicated in Treg function, Treg suppression was not impaired in galectin-9 deficient mice indicating that TIM-3 would exert such function independent of galectin-9 [29].

There are several reports suggesting that TIM-3 has a functional role in T cell exhaustion. We have found that compared to CD8 T cells that harbour the classical exhaustion marker PD-1, the number of TIM-3 expressing T cells is much lower in viremic HIV-1-infected patients as well as in individuals where the virus is suppressed by antiretroviral therapy. Furthermore, exhausted T cells have been described as being CD45RO^+^ [30] and in line with this, the classical exhaustion marker PD-1 was preferentially expressed on CD45RA^−^CD8 T cells, which are CD45RO positive. By contrast, most TIM-3^+^ CD8 cells were found to belong to the CD45RA^+^ subset. The analysis of CD57 expression also suggested differences between PD-1 and TIM-3 expressing T cells: TIM-3 was co-expressed with CD57, whereas the percentage of PD-1 expressing cells was higher in the CD57^−^ subset. Thus, the majority of TIM-3 expressing cells express CD45RA and CD57, indicating that TIM-3 defines a terminally differentiated cell population that is distinct from classical PD-1 expressing exhausted T cells. Jones et al. have also reported that in HIV-1-infected individuals the TIM-3 expressing T cell population is distinct from the PD-1 expressing T cells [10]. They found that the TIM-3 expressing cells are impaired in their ability to produce cytokines, which is in line with a terminally differentiated phenotype of these cells observed in our study. Differences in the amount of TIM-3 expressing T cells in these two studies might result from different staining techniques but also due to the fact that the cohort studied by Jones et al. included a high number of acute/early HIV infections where they observe the highest levels of TIM-3 expressing T cells. In contrast, the cohort studied here comprises chronically infected patients only.

Previous studies have in most cases used the TIM-3 antibody 2E2 - which has been suggested to function as a TIM-3 antagonist – to assess the role of human TIM-3 during T cell activation and exhaustion. We have used Gag and Nef-specific T cells derived from HIV-1-infected individuals as a model system to compare TIM-3 antibody 2E2 and blocking PD-L antibodies regarding their capacity to enhance HIV-1-specific T-cell responses *in vitro*.

In these experiments we found that whereas PD-L blockade significantly enhanced proliferation and IFN-γ production in HIV-1-specific T cells, TIM-3 antibody 2E2 was completely ineffective in this respect (Figure 6; Table 1). In contrast to a previous study we found that TIM-3 antibody 2E2 was also ineffective in enhancing cytokine production of anti-CD3/CD28 stimulated human CD4 cells [20]. Importantly, we also investigated the effect of TIM-3 antibodies during the stimulation of human Th1 polarized human CD4 T cells with allogeneic monocyte derived DC. Despite the high expression of TIM-3 on these T cells TIM-3 antibodies have also been ineffective in these experiments. Taken together our study does not yield any evidence for a role of TIM-3 during human T cell activation processes. Previous reports suggest that TIM-3 exerts its functional role upon interacting with ligands, since TIM-3 antibodies and TIM-3 fusion proteins had similar effects. We have repeatedly screened high quality expression libraries generated from resting and *in vitro* activated human PBMC or human DC [31], [32] with TIM-3 fusion proteins to identify TIM3-ligands. These attempts did not yield TIM-3 binding clones (Figure S5). Although it cannot be ruled out completely that TIM-3 interacts with molecules that were not represented in the cell pools used for screening, it might also indicate that human PBMC and DC do not express TIM-3 ligands.

TIM-1 and TIM-4 bind phosphatidylserine (PtdSer), which is exposed on the surface of apoptotic cells via a conserved binding pocket termed metal ion-dependent ligand binding site (MILIBS) localized on the N-terminal end of their IgV domain [33]. Importantly, human as well as murine TIM-3 molecules also harbour such a motif and DeKruyff et al. have demonstrated that immunoglobulin fusion proteins representing TIM-3 bind to PtdSer in liposomes in a calcium-dependent manner. Furthermore, they found that cells transfected with human TIM-3 bind and phagocytose apoptotic cells [34]. In line with these results it was described that TIM-3-mediated phagocytosis of apoptotic cells is crucially involved in cross-presentation and was linked to peripheral tolerance [35].

Thus it is quite possible that a major functional role of TIM-3 lies in the interaction with apoptotic cells. Engulfment of apoptotic cells by phagocytes can result in potent anti-inflammatory effects and prevent autoimmunity [36]–[38]. TIM-3 is expressed on APC including microglial cells of the CNS [21]. Thus, as already pointed out by DeKruyff et al., enhanced experimental autoimmune encephalomyelitis or reduced transplant tolerance that was observed in mice upon blockade or absence of TIM-3 could be explained by impaired phagocytic activity [34]. Identification of TIM-3 ligands expressed on intact cells would greatly facilitate investigations on potential additional functions for this molecule in immunity.

## Materials and Methods

### Ethics statement

The ethics review board of the General Hospital and the Medical University of Vienna approved collection of blood samples from HIV-1-infected patients and from healthy donors. Written informed consent was obtained from the HIV-1-infected study participants and from the healthy donors prior to blood sampling. All samples were anonymized and research confirmed to the guidelines of the ethics review board of the General Hospital and Medical University of Vienna.

### Cell culture

RPMI-1640 supplemented with 10% FBS, antibiotics and amphotericin was used to culture the mouse thymoma cell line Bw5147 (short designation within this work: Bw cells), T cell stimulator cells and for functional assays with cells derived from healthy donors. Functional assays with cells from HIV-1-infected individuals were done in AIM-V medium (Invitrogen, Carlsbad, CA) in presence of costimulatory antibodies to CD28 (#28.2, Biolegend, San Diego, CA) and CD49d (# L25; BD-Pharmingen, San Diego, CA; final concentration 0.1 µg/ml each).

### Retroviral transduction and binding studies

The coding sequences of human and murine galectin-9 and human and mouse TIM-3 were PCR-amplified and cloned into the retroviral expression vector pCJK2 [15]. Sequence analysis was performed and constructs that contained sequences that were identical to the coding sequences of Genbank entries: NM_032782 (human TIM-3), BC_106851 (murine TIM-3), NM_001159301 (human galectin-9) and NM_002308 (murine galectin-9) were selected for further use. Bw-transductants expressing these molecules and T cell stimulator cells expressing human galectin-9 were generated using previously described retroviral transduction protocols [15]. Expression of these molecules was confirmed using goat-anti-human galectin-9, goat-anti-mouse galectin-9 (both from R&D Systems, Minneapolis, MN), mAbs to human (#F38-2E2, short designation 2E2) or mouse TIM-3 (#B8.2C12; both from Biolegend). Bound antibodies were detected using appropriate PE-conjugated secondary reagents from JacksonImmunoResearch (West Grove, PA). Mouse and human TIM-3 immunoglobulin (Ig) fusion proteins, human ILT5 Ig, human TROP2 Ig, human TREML2 Ig, mouse CTLA4 Ig fusion proteins - all expressed in NSO-cells – and recombinant human (rh) and recombinant mouse (rm) galectin-9 were purchased from R&D systems. Human IgG preparations were bought from JacksonImmunoResearch. In additional experiments immunoglobulin fusion proteins representing the extra-cellular domains of human TIM-3 or human EpCam or B7-H3 produced in our laboratory were used. These fusion proteins were generated and expressed in 293T cells using previously described methods [39]. In flow cytometry experiments the binding of Ig-fusion proteins was detected using goat-anti-human-IgG-Fc-PE antibodies (Jackson ImmunoResearch). Rh-galectin-9 and rm-galectin-9 were labelled using Biotin-X-NHS (Sigma-Aldrich Chemie GmbH, Taufkirchen, Germany). Binding of biotinylated galectin-9 was detected using Strepdavidin-PE (BD Pharmingen). Flow cytometric analysis was done using a FACSCalibur flow cytometer supported by CELLQUEST software (Becton Dickinson). Fluorescence intensity is shown on a standard logarithmic scale. For ELISA-based binding studies, recombinant galectin-9 was reconstituted in PBS and immobilized overnight at room temperature on high-protein binding ELISA plates (Maxisorp; NUNC/Thermofisher Fremont, CA; coating concentration 0.5 µg/ml and 1 µg/ml for human and murine galectin-9, respectively). The plate was washed and blocked with PBS-0.5% BSA (1 h at 37°C). Immunoglobulin fusion proteins were added at the indicated concentrations in PBS-0.5% BSA and incubated for 2 hours at 37°C. The plate was washed and HRP-labelled goat-anti-human IgG antibodies (Fc-specific; JacksonImmunoResearch, diluted 1∶800) were added and incubated for 2 hours at 37°C. Plates were washed and developed using ABTS solution (Roche Applied Science, Mannheim, Germany). Following 15 min incubation, the O.D. 405 nm was determined using 650 nm as reference wave length.

### Functional assays with T cells of healthy donors

T cell activation experiments using the system of T cell stimulator cells were done as previously described [15]. Briefly, irradiated (6000 rad) T cell stimulator cells (2×10^4^/well) were co-cultured with 1×10^5^ T cells, and following 48 h of stimulation, cell culture supernatant was harvested for cytokine measurement and methyl-^3^[H]-thymidine was added to the cultures. Expression of membrane-bound anti-CD3 antibody fragments on the T cell stimulator cells was detected via DyLight-649-conjugated goat antibodies to mouse IgG-H+L specific (Jackson ImmunoResearch). Isolation of human T cells and monocytes and generation of immature and mature monocyte-derived DC was done as previously described [40]. For T cell proliferation assays with plate-bound antibodies, antibodies to CD3 (#OKT3, ADG, Kaumberg, Austria) and CD28 (#28.2, Biolegend) were immobilized for 4–5 h 37°C at the indicated final concentrations. Following two washing steps, 1×10^5^ T cells/well were added and cultured for 48 h in the presence of soluble TIM-3 antibodies or isotype control antibodies (both Biolegend, final concentration: 5 µg/ml) as previously described [20]. For Th1 polarisation MNCs were activated with antiCD3/antiCD28 coated beads (Dynabeads, Invitrogen) and IL-2 (300 U/ml; PeproTech Inc. Rocky Hill, NJ) for two days. Subsequently, cells were washed and IL-12 (25 ng/ml; PeproTech Inc) and IL-2 (100 U/ml) were added for 3 days. For DC stimulation assays human T cells (1×10^5^/well) were co-cultured with allogeneic immature or mature human DC at the indicated cell numbers for 5 days in presence of the indicated antibodies (final concentration 10 µg/ml). To assess T cell proliferation methyl-^3^[H]-thymidine (final concentration: 0.025 mCi; Perkin Elmer/New England Nuclear Corporation, Wellesley, MA) was added for the last 18 hours of culture. Methyl-^3^[H]-thymidine uptake was measured as described [41].

### Experiments with T cells derived from HIV-1-infected individuals

Blood samples from patients infected with HIV-1 were obtained during routine check-ups at the outpatient clinic of the HIV-unit of the Department of Dermatology, Division of Immunology, Allergy and Infectious Diseases, Medical University of Vienna, Austria.

Patients were referred to as either virologically suppressed when on ART (antiretroviral therapy) with a virus load below the limit of quantification (20 copies/ml) for at least two consecutive controls or at least 3 months or viremic (ART naïve; viral load above 1000 copies/ml). Mean duration of ART was 61 (±52) months. Patients with ongoing opportunistic infections or tumours or any other obvious acute medical conditions were excluded from the analysis.

For analysis of TIM-3 and PD-1 expression on T cells of HIV-1-infected and healthy donors, whole blood samples were stained with TIM-3-PE, PD-1-PE or a PE-labelled isotype control (all from Biolegend). For TIM-3/PD-1 costaining, a PD-1-APC from BD Pharmingen was used. Cells were counterstained with CD8-APC, CD4-PerCP, and CD45RA-FITC or CD57-FITC (all from BD Pharmingen). Subsequently, cells were washed and treated with ADG lysis (Kaumberg, Austria).

PBMC of HIV-1-infected individuals were isolated using density gradient centrifugation and stimulated for 7 days in triplicates in flat-bottom 96-well plates with overlapping 15-mer HIV-1 Clade B Gag and Nef peptide pools (generously provided by the National Institutes of Health AIDS Research and Reference Reagent Program; final concentration 20 µg/ml). All stimulation experiments were performed in absence of antibodies or in presence of isotype control mAb (#MOPC-21), a combination of PD-L1 mAb (#29E.2A3) and PD-L2 mAb (# 24F.10C12), or TIM-3 mAb (#F38-2E2). mAbs (purchased from Biolegend in functional grade quality) were used at a final concentration of 10 µg/ml. Following 6 days of stimulation, culture supernatants were harvested for INF-γ measurement and subsequently methyl-^3^[H]-thymidine (final concentration: 0.025 mCi) was added and methyl-^3^[H]-thymidine incorporation was determined as described above.

In an additional set of experiments T cell proliferation was assessed by CFSE staining. CFSE labelling was performed as described previously [42] and stimulation of CFSE-labelled PBMC was done for 7 days as described above with the exception that 24 well plates were used. Cells were harvested, stained for CD8 expression and analyzed by FACS as described. In both types of experiments mock stimulation cultures without HIV-1 peptides were set up in parallel for each donor to assess specific response to Gag/Nef peptides (at least 2-fold greater than mock treated samples).

### Cytokine measurement

Supernatants from T cell activation experiments were harvested prior to addition of methyl-^3^[H]-thymidine, and pooled from triplicate wells and used for measurement of IL-2, IL-10, IL-13, IL-17 and IFN-γ. Supernatants from stimulation cultures of T cells derived from HIV-1-infected individuals were harvested on day 6 and used for measurement of IFN-γ. All measurements were performed in duplicates using the Luminex System 100 (Luminex, Texas, USA).

### Statistical analyses

Two-tailed Student's-t test was used to assess significance. Error bars indicate the SD.

## Supporting Information

Figure S1 A) Human T cells derived from healthy donors were co-cultured with T cell stimulator cells expressing human galectin-9, PD-L1, CD80, CD86 or no human costimulatory molecule (control). Methyl-^3^[H]-thymidine uptake was assessed following 3 days of co-culture (cpm, counts per minute). The results of triplicate measurement of one experiment representative for eight experiments are shown. n.s.: not significant. B) T cells were co-cultured with control stimulator cells and stimulator cells expressing FasL or galectin-9. Following 3 days of stimulation annexin-V/propidium iodide staining was performed.
(EPS)

Figure S2 A) Bw cells transduced to express human CD28 and parental Bw cells (Bw control) were stained with CD28 antibodies or with immunoglobulin fusion proteins representing the extra-cellular domains of human CD80 (CD80-Ig). B) Bw control cells (open histograms) or Bw cells transduced to express human TIM-3 (grey histograms) were probed with human galectin-9 at the indicated concentrations. Bound galectin-9 was detected with an anti-galectin-9 antibody followed by PE-labelled donkey-anti-goat antibodies. C) Recombinant human galectin-9 (0.5 µg/ml) was immobilized on ELISA plates and probed with two different human TIM-3-Ig preparations. TIM-3-Ig and mCTLA4-Ig (as control) from R&D, and TIM-3-Ig and EpCam-Ig (as control) produced in house were used at the indicated concentrations.
(EPS)

Figure S3 Parental Bw cells (Bw control) and Bw cells transduced to express PD-1 or TIM-3 were stained with PE-labelled isotype control antibody (IgG-PE) or PD-1-PE and TIM-3-PE as indicated and analyzed by flow cytometry.
(EPS)

Figure S4 A) Percentage of TIM-3 positive cells in the CD45RA^+^CD8 T cell subset from suppressed (S) and viremic (V) patients and from healthy individuals (H) are shown. Bars indicate median percentage. B) Co-expression of TIM-3 and PD-1 on total CD8 (upper right), CD8/CD45RA^+^ (middle right) and CD8/CD45RA^−^ T cells from a viremic patient.
(EPS)

Figure S5 Comprehensive retroviral cDNA expression libraries generated from immature and mature dendritic cells (DC) and freshly isolated and *in vitro* activated human PBMC were co-expressed in Bw cells. Cells were probed with immunoglobulin (Ig) fusion proteins representing the extra-cellular domains of human CTLA-4 (“Abatacept”), human PD-1 or human TIM-3. Horse serum was used to block Fc-receptor binding. Bound immunoglobulin fusion proteins were detected with PE-conjugated goat-anti human IgG (Fc-specific) antibodies. The number and percentage of cells in the sorting gate are shown. Sorted cells were expanded and subjected to additional rounds of sorting. This yielded CTLA4-Ig and PD-1-Ig reactive cells whereas no TIM-3-Ig reactive cells were obtained (data not shown). Several similar library sorting experiments were performed with the same outcome.
(EPS)
